# Individual Microparticle Manipulation Using Combined Electroosmosis and Dielectrophoresis through a Si_3_N_4_ Film with a Single Micropore

**DOI:** 10.3390/mi12121578

**Published:** 2021-12-18

**Authors:** Chenang Lyu, Leo Lou, Matthew J. Powell-Palm, Gideon Ukpai, Xing Li, Boris Rubinsky

**Affiliations:** 1 Department of Food Science and Technology, Shanghai Jiao Tong University, Shanghai 200240, China; 2 Department of Mechanical Engineering, University of California Berkeley, Berkeley, CA 94720, USA; mattpowellpalm@gmail.com (M.J.P.-P.); ukpai.gideon@gmail.com (G.U.); lixinglx918@gmail.com (X.L.); brubinsky@gmail.com (B.R.); 3 Department of Bioengineering, University of California Berkeley, Berkeley, CA 94720, USA; taolou@berkeley.edu; 4 Electronic Countermeasure Institute, National University of Defense Technology, Hefei 230037, China

**Keywords:** microparticle manipulation, electro osmosis, dielectrophoresis, dielectric film, micropore

## Abstract

Porous dielectric membranes that perform insulator-based dielectrophoresis or electroosmotic pumping are commonly used in microchip technologies. However, there are few fundamental studies on the electrokinetic flow patterns of single microparticles around a single micropore in a thin dielectric film. Such a study would provide fundamental insights into the electrokinetic phenomena around a micropore, with practical applications regarding the manipulation of single cells and microparticles by focused electric fields. We have fabricated a device around a silicon nitride film with a single micropore (2–4 µm in diameter) which has the ability to locally focus electric fields on the micropore. Single microscale polystyrene beads were used to study the electrokinetic flow patterns. A mathematical model was developed to support the experimental study and evaluate the electric field distribution, fluid motion, and bead trajectories. Good agreement was found between the mathematic model and the experimental data. We show that the combination of electroosmotic flow and dielectrophoretic force induced by direct current through a single micropore can be used to trap, agglomerate, and repel microparticles around a single micropore without an external pump. The scale of our system is practically relevant for the manipulation of single mammalian cells, and we anticipate that our single-micropore approach will be directly employable in applications ranging from fundamental single cell analyses to high-precision single cell electroporation or cell fusion.

## 1. Introduction

The ability to precisely manipulate single microparticles and cells is important in many micro- and nano- scale fluidic devices [1,2,3]. Electrokinetic transport based on electroosmosis (EO), dielectrophoresis (DEP), and electrophoresis (EP) is a widely used manipulation technique in microfluidics due to its implicit simplicity, low cost, and ease of fabrication [4,5,6].

EO is induced by ionic cloud migration in response to electric fields that are applied tangentially to an electrode surface [7]. Electroosmotic micropumps (EOP) can create constant pulse-free flows in low Reynolds number flow (in which a traditional external pump system may work inefficiently) without the requirement of moving parts [4]. The flow rates and pumping pressure of EOPs have a quick and precise response to electric input, making the suitable for use with microanalysis systems [6].

DEP occurs when a polarizable particle is suspended in a spatially nonuniform electric field [8]. If the particle moves in the direction of an increasing electric field, the behavior is referred to as positive DEP (pDEP), while if it moves away from the high electric field regions, it is known as negative DEP (nDEP). Dielectrophoresis can be used to manipulate, transport, separate, and sort different types of particles based on the frequency-dependent relative polarizabilities of the particle and medium [9,10,11]. To date, the vast majority of DEP-based systems can be classified as electrode-based dielectrophoresis (eDEP), insulator-based dielectrophoresis (iDEP) [12], and light induced DEP [13,14,15]. In iDEP chips, where the gradient of the electric field is formed by geometrical constrictions within insulating substrates instead of metallic microelectrodes, the electrodes are positioned remotely and do not contact the particles or cells directly.

The most common designs for iDEP feature two-dimensional (2D) microchannels connected to inlet and outlet liquid reservoirs and exposed to with nonuniform electric fields. Three-dimensional (3D) variants have garnered increasing attention due to their lower voltage requirements, reduced Joule heating, and superior extensibility [12]. One critical configuration of 3D iDEP systems makes use of porous membranes, as the insulating structure. Several relevant studies have reported the trapping and agglomeration of a wide array of particles, ranging from biomolecules to cells, using dielectrophoresis and porous membranes. For example, Kovarik and Jacobson employed a track-etched nanomembrane with conical pores (130 nm in diameter at the tip, 1 µm in diameter at the base, and 10 µm long) for the trapping of polystyrene particles and Caulobacter crescentus cells [16]. Cho et al. report dielectrophoretic trapping of *E. coli* cells in a membrane-based system (a SU-8 photoresist with a thickness of 200 µm) [17].

However, despite significant interest in porous membrane-based iDEP techniques, few prior studies have addressed the fundamental local electrokinetic behavior around a single micropore in a thin dielectric film. In this study, we fabricate a silicon nitride film with a single micropore (2–4 µm in diameter), ensembled in an axisymmetric 3D chamber which locally focuses the electric field on the micropore. We have used this device to study the flow pattern of single microscale polystyrene beads in relation to an electric field focusing micropore. Our experiments demonstrate that the combination of electroosmotic flow and DEP forces induced by direct current has significant potential as a means to trap, agglomerate, repel, and rotate the beads without an external pump. A finite element (FEM) based mathematical model was developed in support of the experimental study, to predict the particle movements around a single micropore as a result of electroosmotic flow and DEP forces. We find that the mathematical model can capture the experimental results with high fidelity. The scale of our system is practically relevant for the manipulation of single mammalian cells, and we anticipate that our single-micropore approach will be directly employable in applications ranging from fundamental single-cell analyses, to high-precision single cell electroporation, or cell fusion.

## 2. Materials and Methods

### 2.1. Micro-Pore Chip Fabrication

**Figure 1:**
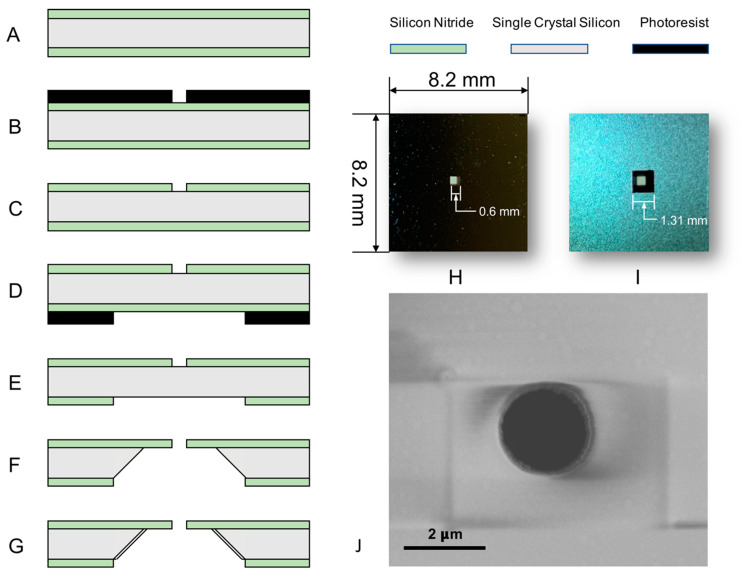
(**A**–**G**) Fabrication process for the dielectric film with a single micropore. (**H**,**I**) The pictures of the polished film side and unpolished well side of the chip. (**J**) The scanning electron microscope (SEM) picture of a typical micropore.

The experiments were performed on a dielectric film with a single-micropore. Low-stress silicon nitride (Si_3_N_4_) was chosen as the film material because it is a well-established mask material for typical silicon etchants, and is optically translucent under microscopy [18,19]. Figure 1 shows the step-by-step fabrication process of the dielectric film with the micropore. This dielectric film is the core component of the chip used in this study. The process begins with a <100> n-type, single-side polished, single-crystal silicon (SCS) wafer. Low pressure chemical vapor deposition (LPCVD) was used to deposit a 1.0 μm, low stress silicon nitride layer on each side of the wafer (Figure 1A). Then, the photoresist was spun on the polished side of the wafer, and the micropore was patterned with a mask aligner (Karl Suss MA6 Mask Aligner) (Figure 1B). The patterned micropore was etched through the silicon nitride film by a plasma etcher (Lam6 Oxide Rainbow Etcher) (Figure 1C). Typical diameters of the micropore range from 2.2 to 4 μm (Figure 1J). After stripping off the photoresist left on the polished side of the wafer, a new layer of photoresist was deposited and patterned on the unpolished side of the wafer to open a window (1.31 mm × 1.31 mm) for the Potassium Hydroxide (KOH) etch step (Figure 1D). Then, the unpolished side window was opened with plasma etching (Figure 1E). Once the micropore and the unpolished side window were patterned on the silicon nitride layer, the wafers were dipped into a 24% *v*/*w* KOH solution at 80 °C in order to completely etch the exposed silicon (Figure 1F). A well was formed originating from the unpolished side and terminating at the LSN film. Finally, a 0.1 μm silicon dioxide (SiO2) layer, which serves as the electrically insulating layer between the silicon wafer and liquid, was thermally grown over the exposed silicon on the well’s side wall after the KOH etching (Figure 1G) [18]. Figure 1H,I shows the pictures of the polished side and unpolished side of the diced chip. Figure 1J shows the scanning electron microscope (SEM) picture of a typical micropore.

### 2.2. Experimental Set-Up

**Figure 2:**
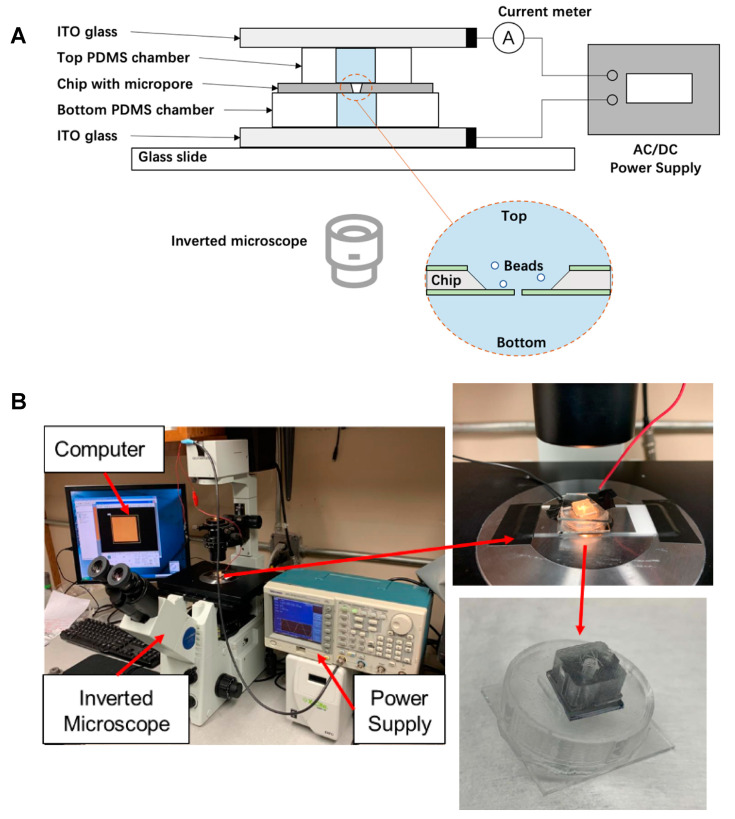
(**A**) Schematic illustration of the experimental setup. (**B**) The picture of experiment setting.

Figure 2A shows a schematic illustration of the experimental setup, and Figure 2B shows the corresponding pictures. The chip (polished film side face down) is sandwiched between two PDMS hollow discs, and sealed with indium tin oxide (ITO) coated glasses (Nanocs, NY, USA) at the ends of the discs, forming two chambers with a diameter of 3 mm and a height of 2 mm. The bottom chamber is filled with deionized water and the top chamber is filled with an aqueous suspension of diluted polystyrene (PS) beads (0.1% w/w, 10 μm in diameter, Sigma-Aldrich Co. St. Louis, MO, USA). The conductivities of the top and bottom liquid, measured by a conductivity meter (Elite PCTS tester, Thermo Fisher Scientific, Waltham, MA, USA), were found to be similar (2.3 × 10^−4^ S/m). The whole device is placed on an insulated slide and observed on an inverted microscope. The ITO coated glasses were connected to a Waveform generator (Model WW1072, Tabor Electronics) as the power supply. A mounted microscope camera, connected to a computer, recorded the movement of the particles during the experiment. We employed DC current; however, no appreciable electrolytic gas generation was observed during the experiment, probably because of the low ionic content of the deionize water and the short duration of the experiments. The measured current is about 139 nA when 10 V was applied.

### 2.3. Theoretical Estimation for the Movement of the Beads

**Figure 3:**
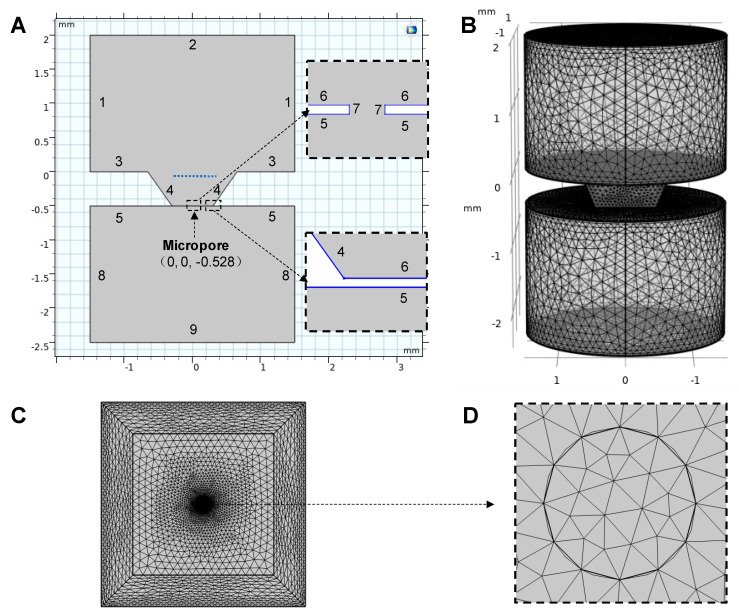
(**A**) The setting for the boundary. (**B**) The mesh (free tetrahedral) distribution of the whole model. (**C**) The bottom view of the mesh distribution on the film. (**D**) The magnification of the mesh near the micropore.

To better understand the movement of the PS beads during experimentation, we developed a three-dimensional cross-section FEM model using COMSOL Multiphysics. The electric field distribution, fluid flow, and particle trajectories in the liquid were calculated. As Figure 3A shows, the Si_3_N_4_ film (consisting of boundaries 5, 6, 7) separates the top chamber (well) from the bottom chamber (under the film), which were both filled with DI water. The two chambers were connected via the micropore, which in the model was assigned a diameter of 4 μm. The parameters used in this study are listed in Table S1 of the Supplementary Materials. Figure 3B shows the FEM model mesh. Figure 3C shows the mesh of the film from bottom view and Figure 3D shows the magnification of mesh around the micropore. The position of the pore in the model is (0 mm, 0 mm, −0.528 mm).

#### 2.3.1. Electrical Field Distribution

The electric field distribution in the geometry of Figure 3A was solved for two boundary conditions. Electrical potentials of either 10 V or −10 V were applied on the surface of the top electrode (boundary 2). Ground was set on boundaries marked 10. The remaining boundaries were insulated. The conductivity of the DI water used in the experiment was measured to be 2.3 × 10^−4^ S/m, and this value was also used in the mathematical model. The governing equation is the conservation of current:

(1) ∇·J=0

where ∇·() is the divergence operator and J represents the local current density vector. The current density only has the conductive component at steady state and is given by:

(2) $$J=\left(\sigma +\varepsilon_{0}\varepsilon_{r}\frac{\partial }{\partial_{t}}\right)E$$

E represents the local electric field, σ is the conductivity. The electric field is linked to the potential field, U, by the relationship:

(3) E=−∇U

The field equation is solved for the geometry and electrode locations in Figure 3A.

#### 2.3.2. The Fluid Flow Model

The fluid flow was also calculated. The fluid motion is governed by the incompressible Navier-Stokes equation:

(4) $$\rho \frac{\partial u}{\partial t}-\nabla \cdot \eta \left(\nabla u+{\left(\nabla u\right)}^{T}\right)+\rho \left(u\cdot \nabla \right)u+\nabla p=0$$

(5) ∇·u=0

Here, η refers to the dynamic viscosity (kg/(m·s)), u  is the velocity (m/s), ρ equals the fluid density (kg/m^3^), and p denotes to the pressure (Pa).

Most solid surfaces in contact with an electrolyte, acquire a surface charge. In response to the spontaneously formed surface charge, ions are accumulating at the liquid-solid interface. Known as an electrical double layer, it forms because of the ions located on the surface face the solution. When an electric field is applied, the electric field generating the electroosmotic flow displaces the charged liquid in the electrical double layer [20]. This scheme imposes a force on the charged solution close to the wall surface, and the fluid starts to flow in the direction of the electric field. The velocity gradients perpendicular to the wall give rise to viscous transport in this direction. In the absence of other forces, the velocity profile eventually become almost uniform in the cross-section perpendicular to the wall [21]. Our model replaces the thin electric double layer with the Helmholtz-Smoluchowski relation between the electroosmotic velocity and the tangential component of the applied electric field [22]:

(6) $$u=\frac{\varepsilon_{w}\zeta_{0}}{\eta }\nabla U$$

In this equation, $\varepsilon_{w}=\varepsilon_{0}\varepsilon_{r}$ denotes the fluid’s electric permittivity (F/m), $\zeta_{0}$ represents the zeta potential at the channel wall (V), and U equals the potential (V). This equation applies to all boundaries except for the entrance and the outlet. 

The electroosmotic velocity condition was applied on the Si_3_N_4_ surface (Boundaries 5, 6, 7 with a zeta potential of −28 mV [23]), the SiO_2_ surface (Boundaries 4 with a zeta potential of −42 mV [23]) and the electrodes’ surfaces (Boundaries 2, 9 with a zeta potential of −100 mV). 

#### 2.3.3. Particle Trajectories

For the particles tracing the fluid flow, multiple particles (10 μm in diameter) were released from a grid position above the pore (Blue dot line in Figure 3A, X range from −0.5 mm to 0.5 mm, Y range from −0.5 mm to 0.5 mm, Z is −0.1 mm) with an initial velocity of zero. We set the boundary condition on the silicon nitrate substrate to be sticky so that if a particle falls onto the film, that particle could no longer move. The particles were subject to Stokes drag forces, dielectrophoresis forces, gravitational forces, and buoyancy forces and electrophoretic forces. The Stokes drag force was governed by the equation:

(7) $$F_{D}=\frac{1}{\tau_{p}}m_{p}\left(u-v\right)$$

where $m_{p}$ is the mass of the particle, u is the velocity of the fluid, v is the velocity of the particle, and $\tau_{p}$ is the particle velocity response time, given by:

(8) $$\tau_{p}=\frac{\rho_{p}d_{p}^{2}}{18\eta }$$

In which $\rho_{p}$ is the density of the particle, $d_{p}$ is the diameter of the particle.

The gravitational and buoyancy forces were calculated by following the equation:

(9) $$F_{g}=m_{p}g\frac{\rho_{p}-\rho }{\rho_{p}}$$

where g is the gravity vector.

The DEP force acting on the PS beads was calculated from the following equations:

(10) $$F_{DEP}=2\pi r_{p}^{3}\varepsilon_{f}real\left(K\left(\omega \right)\right)\nabla {\left|E\right|}^{2}$$

where $r_{p}$ is the radius of PS beads, $\varepsilon_{f}$ is the permittivity of fluid, E is the applied electric field. 

The Clausius-Mossotti factor of PS beads (perfectly spherical particles), K(ω) is a ratio of complex permittivity:

(11) $$K\left(\omega \right)=\frac{{\tilde{\varepsilon }}_{p}-{\tilde{\varepsilon }}_{f}}{{\tilde{\varepsilon }}_{p}+2{\tilde{\varepsilon }}_{f}}$$

(12) $$\tilde{\varepsilon }=\varepsilon -\frac{i\sigma }{\omega }$$

where *ω* is the frequency, *ε* is the dielectric constant, and σ is the electrical conductivity of the medium.

When no frequency component is involved, DC-DEP can be estimated as the residual of this factor when frequency goes to zero:

(13) $$\underset{\omega \to 0}{\lim}\;K\left(\omega \right)=\frac{\sigma_{p}-\sigma_{f}}{\sigma_{p}+2\sigma_{f}}$$

where $\sigma_{p}$ and $\sigma_{f}$ is the real conductivity of the particle and fluid respectively [24]. In our experiment, $\sigma_{p}$ is much smaller then $\sigma_{f}$, which will induce negative dielectrophoresis.

The electrophoretic forces were calculated by following the equation:

(14) $$F_{EP}=qE$$

The zeta potential of the PS beads was set to be −40 mV [25].

## 3. Results

### 3.1. Theoretical Estimations

#### 3.1.1. Electrical Field Distribution

**Figure 4:**
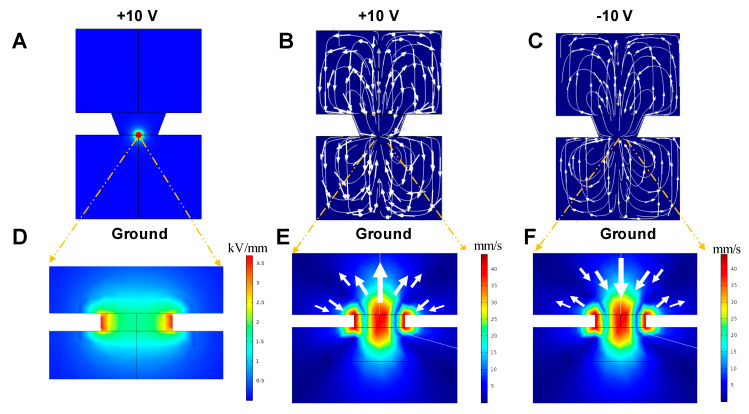
(**A**) The electric field distribution in the DI water. (**D**) The magnification of the electrical field distribution near the micropore. (**B**,**C**) The steady state fluid streamlines around the chip when the bottom electrode is grounded and the top electrode is +10 V or −10 V, respectively. (**E**,**F**) The corresponding magnified velocity field near the pore for figures (**A**,**B**), respectively. The arrow indicates the velocity of the fluid.

Figure 4A shows the electric field distribution in DI water when +10 V was applied to the top electrode surface (boundary 2) and the bottom surface was grounded. Higher magnification of the electric fields is shown in the right panel of Figure 4D. It shows a typical electric field distribution in the fluid near the pore, which has a maximum electric field of 37.3 kV/cm at the edge of the pore. The electric fields decrease radially from the edge of the pore (the point of singularity) outward, similar in effect to those featured in our previous studies [26,27]. In other words, the micropore locally focuses the electric field.

#### 3.1.2. Motion of the Fluid

Figure 4B,C show the flow velocity magnitude and the streamline of the flow in the top and bottom chambers when +10 V and −10 V is applied on the surface of the top electrode at steady state, respectively. The bottom surface is grounded in both cases. The arrow indicates the direction of the fluid flow. The electroosmotic flow that circulates near the pore is much larger than in the other parts of the system. Detailed flow velocity magnitude distribution near the pore is shown in Figure 4E,F. As Figure 4B reveals, the fluid above the dielectric film flows from the sides towards the pore’s edge but is then pushed vertically upwards as it approaches the center of the pore. The fluid motion in the bottom chamber is in an opposite direction to that in the top chamber. When a voltage of −10 V was applied on the top surface (Figure 4C), the fluid above the center of the pore is pulled downward to the pore and then flows from the pore’s edge to the sides. A detailed look at the fluid motion at the edge of the pore (Figure 4E,F) indicates that the maximum velocity is found at the edge of the pore, corresponding to the location of the maximum electric field in Figure 4B,C. According to our calculation, the Joule heating induced temperature rise is only 0.2 °C when +10 V was applied, so flow induced by Joule heating was determined to be negligible compared to the electroosmotic flow.

#### 3.1.3. Particle Trajectories

**Figure 5:**
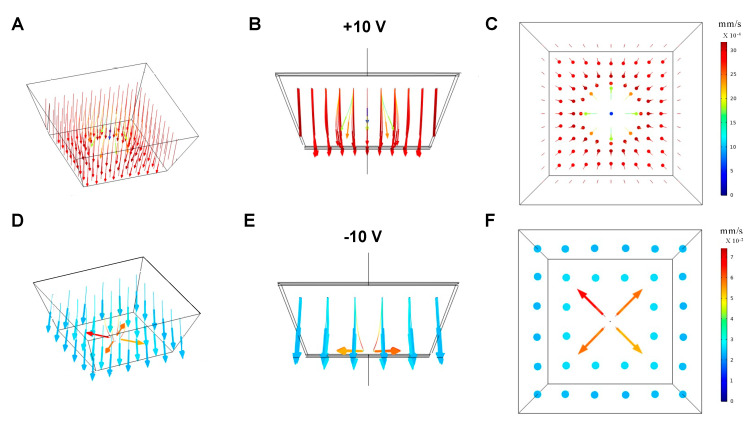
(**A**) Particle evolution when +10 V is applied to the top electrode. Side view (**B**) and top view (**C**) of the particle trajectories. (**D**) Particle evolution when −10 V is applied to the top electrode. Side view (**E**) and top view (**F**) of particle trajectories.

Figure 5 shows results simulating the movement of the 10 μm PS beads released from the grid above the pore. The particles were driven by drag forces, DEP forces, gravity forces, and buoyancy forces. Figure 5A–C show that when the top surface electrode potential is +10 V from different time point, the particles directly above the pore are repelled from the pore by drag force due to the upward fluid flow from the pore center. However, The particles around the pore (not directly above) were attracted to the pore (Figure 5C). The flow was reversed when a potential of −10 V was applied to the top surface electrode. As Figure 5D–F show, particles directly above the pore were pulled towards the pore by the downward fluid to the pore center. However, as particles approach the pore, they are repelled (Figure 5F).

#### 3.1.4. The Forces Applied on the Particles

**Figure 6:**
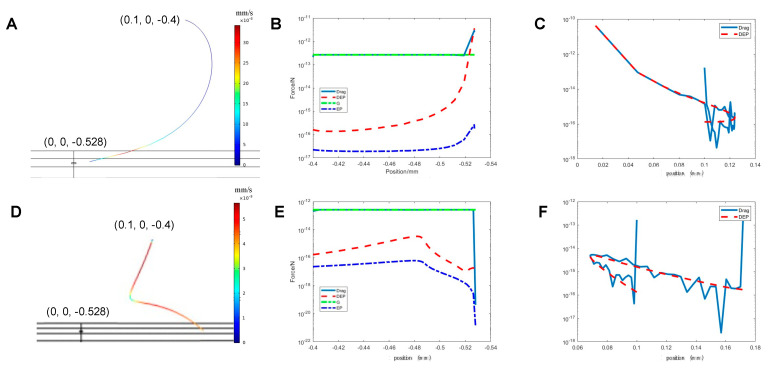
(**A**) The trajectory of the particle released from (0.1 mm, 0 mm, 0.4 mm) with the top surface electrode charged with +10 V. (**B**,**C**) The z- and x-components of DEP force, drag force, and force of gravity when +10 V is applied. (**D**) The trajectory of the particle released from (0.1 mm, 0 mm, 0.4 mm) with the top surface electrode charged with −10 V. (**E**,**F**) The z-component and x-component of DEP force, drag force, EP force, and force of gravity when −10 V is applied.

In our mathematical model, the particles in the solution were affected by drag forces, DEP forces, gravity, and buoyancy forces, and electrophoretic forces. The surface charge of the particles is dependent on various parameters such as the conductivity and pH of the solution [28]. The electrophoretic force could be ignored when compared to the drag force and DEP force (Figure 6B,E). To better understand the particle flow pattern evolution and the forces experienced by single microparticle in the single pore configuration we show two typical results of local force and flow pattern in Figure 6.

Figure 6 shows the particle’s trajectory and its experienced forces when it is released from 0.1 mm, 0 mm, or −0.4 mm with the electrode on the top having a potential of +10 V (Figure 6A–C) and −10 V (Figure 6D–F). The color of the trajectory shows the velocity of the particle. In Figure 6A, the particle is first pushed away from the *x* axis and then dragged back when the x position is 0.12 mm. Next, it accelerates towards the pore and then decelerates rapidly near the pore until it stops. Figure 6B shows the magnitude of z-component of the drag force, DEP force and force of gravity affecting the particle during the whole processing, as a function of Z position. Figure 6C shows the shows the magnitude of z-component of the drag force and DEP force as a function of X position. Figure 6B,C shows that when the particle is far away from the pore, the nDEP force is negligible compared to the drag force and force of gravity, which means the particle’s trajectory is mainly affected by the electroosmotic flow, gravity, and buoyancy forces. However, when the particle approaches the pore, the nDEP force increased exponentially with distance from the pore. The nDEP has an opposite direction with the drag force and finally makes the particle stop. When we released the particle from the same position but reversed the current (Figure 6D–F), the particle was first attracted to the pore in the direction of the flow, and then pushed away when it reached the position of 0.075 mm, 0 mm, −0.482 mm. Both the drag force and nDEP force pushed the particle away close to this point. The complex pattern of forces and flows was evident from these calculations. However, it is also obvious that the voltages imposed as boundary conditions and the initial locations of particles can be used to precisely place microparticles relative to the single micropore.

### 3.2. Experimental Results

**Figure 7:**
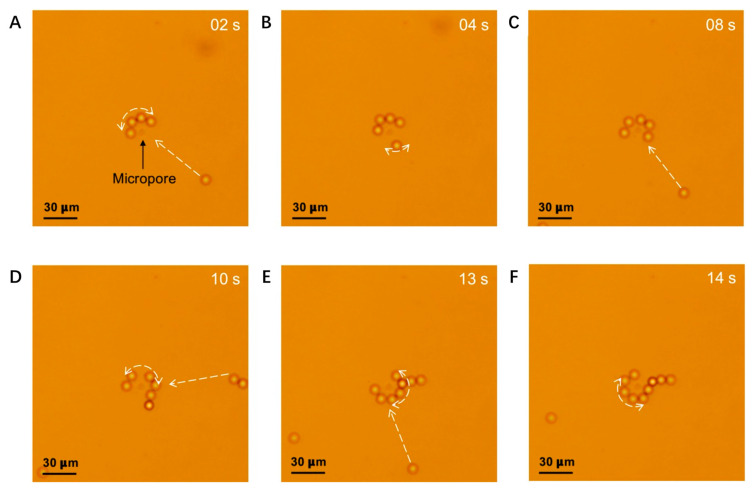
Trajectory of PS beads when +10 V is applied. Images (**A**–**F**) were extracted at t = 2, 4, 8, 10, 13, 14 s from the Video S1. The beads are attracted to the pore in succession and form a ring near the pore. The white dashed arrows represent the track of the beads.

The experimental study allowed us to continuously track the motion of the particles (see Supplementary Materials SV1). We analyzed the recorded trajectories of the PS beads during the experiment and compared this to the mathematical model. Figure 7 shows the PS bead trajectories under the microscope when the beads were on the film initially and +10 V was applied on the top electrodes. Images were extracted at t = 2, 4, 8, 10, 13, 14 s from Video S1. The beads were attracted to the pore in succession, initially increasing in velocity before slowing down to a halt at the equilibrium position. There they form a ring around the pore, which was the same as the result of the FEM mathematical model (Figure 5 and Figure 6). The PS beads could not get closer to the pore, and as such, did not seal it, because of the strong nDEP force. This phenomenon suggests that this approach may have significant potential for the continuous aggregation of microparticles, because the particles were simultaneously robustly captured and prevented from blocking the pore, whereas with a conventional hydrodynamic method (pump), agglomeration stops after the pore is sealed.

**Figure 8:**
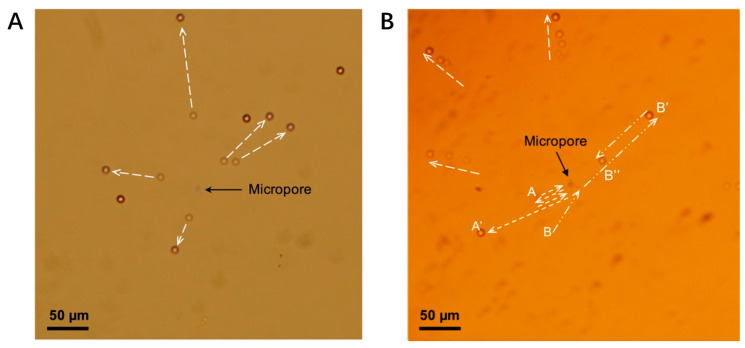
(**A**) Double-exposure pictures showing the particles’ initial position and final position when −10 V was applied to the top electrode. The particles already sitting on the film were repelled from the pore. The dashed arrow represents the trajectory of the PS beads. (**B**) Multi-exposure pictures showing the trajectories of particles A and B (A-A’, B-B’-B’’) when −10 V was applied. The particles A and B, which were still floating in the fluid, were oscillating around the pore.

When the potential of the top electrode was switched to −10 V, the particles already in contact with the film were repelled away from the pore (Figure 8A). This result verified the predictions in Figure 5. Upon switching the potential back to +10 V, the particles were once again attracted to the pore. Thus, by manipulating the magnitude and direction of the applied electric field, the beads could be moved to precise radial positions.

It was interesting to observe that when the particles remained floating in the fluid (Figure 8B), they were observed to rotate around the pore. As the beads rotated in the x–z plane, perpendicular to the film, they appeared to be oscillating around the pore on the x–y plane (A-A’, B-B’-B’’). In these experiments, a portion of the bead trajectories became out of focus as they moved beyond the depth of the field. The beads continued rotating until they came into contact with the film and stopped at the equilibrium position, as Figure 5 depicts.

## 4. Discussion

A simple experimental technique supported by a mathematical model was used to develop a fundamental understanding of the flow patterns of microscale PS beads in a fluid flow affected by a focused DC electric field around a single micropore on a dielectric Si_3_N_4_ film. Both the experimental and mathematical analysis gave fundamental insights into the flow pattern of single noncharged microparticles around a single electric field focusing micropore. A wealth of interesting behavior was observed. It was particularly interesting to observe that particles aggregate near a point of force equilibrium on the dielectric field around the pore. From a practical point of view, our results suggest that in a single micropore configuration the combined effects of DC electroosmotic flow and dielectrophoresis can be utilized to predictably translate, capture, or aggregate small microparticles. This technique eliminates the need for external hydrodynamic resources (such as a pump), and requires no moving parts. Furthermore, the requisite electrodes can be mounted at relatively large distances from the particles in question, a noted strength of iDEP-based techniques.

The technique is also uniquely immune to the blockages that can hinder traditional hydrodynamic techniques, as the competing forces acting upon the particle hold it in stable mechanical equilibrium a finite distance from the micropore itself, preventing particles from blocking the pore and disrupting electrical continuity between the top and bottom of the chip. This phenomenon suggests that this technique may be equally suitable for both the manipulation and trapping of a single particle and the continuous uninterrupted aggregation of particles, which in turn may be sortable based on their dielectric properties [27].

Perhaps most importantly, the complex competing electrokinetic effects play in this system are reliably captured by our mathematical modeling, opening the door to robust optimization of particle manipulation parameters based on desired applications. The experiments presented herein demonstrate that a wide suite of manipulations may be achieved by the strategic combination of dielectrophoresis and DC electroosmotic flow, including attraction or repulsion of particles by control of the direct current direction when the particles are on the film, rotation of floating particles, and stable trapping or aggregation of particles at a predictable equilibrium distance from the micropore. While this study presents only proof-of-concept experimental validation of these phenomena, the modeling principles developed herein can be employed in order to further refine specific experimental manipulations as desired.

The disadvantages of the present chip design include the fact that particles that stick to the film cannot be displaced by the electroosmotic flow once they are in contact with the film (Figure 8A), and the somewhat limited manipulation distance, a consequence of the decreasing strength of both electroosmotic flow and DEP force with increasing distance from the pore. Therefore, a microfluidics channel will be needed in future iterations of the chip to limit the distribution of particles.

In future, alternative current (AC) with different frequencies will be applied to enhance the controllability of the DEP force, potentially enabling selective attraction or repulsion of different particles. Different fluids and particles must also be tested with the chip in order to better understand how the motion of the particles may vary under differing conditions, and how the electrical properties of the particle itself may alter its responses to electrokinetic stimuli.

Based on its unique design and high degree of mathematical predictability, we anticipate that this chip design may have significant potential for biological applications, including single-cell electroporation (in which a single cell can be captured on one side of the film, electroporated, and then induced with DNA or a protein from the other side of the film), cell fusion, or any manner of fundamental single-cell analyses.

## Supplementary Materials

The following are available online at https://www.mdpi.com/article/10.3390/mi12121578/s1, Table S1: The parameters used in the FEM model, Video S1: The motion of the particles.
