# Supplementary material for: Individual Microparticle Manipulation Using Combined Electroosmosis and Dielectrophoresis through a Si3N4 Film with a Single Micropore
Source: Micromachines (Basel). 2021 Dec 18;12(12):1578. doi: 10.3390/mi12121578 (PMC8708253; doi:10.3390/mi12121578)
Supplement: Supplementary file 1 [file micromachines-12-01578-s001.zip › Table S1.pdf]

Table S1 The parameters used in the FEM model

Parameters	Value
The thickness of the Si_3N_4 film	1 um
The radium of the hole	2 um
The thickness of the chip	528 um
The radium of the chamber	1500 um
The height of the chamber	2000 um
The width of the top window	1310 um
The conductivity of the deionized water	2.3e-4 S/m
The permittivity of the deionized water	78
The conductivity of the particle	1e-6 S/m
The permittivity of the particle	2.5
The diameter of the particle	10 um
The density of the particle	1050 kg/m^3
